# Epigenetic‐Based Mechanisms of Osteoblast Suppression in Multiple Myeloma Bone Disease

**DOI:** 10.1002/jbm4.10183

**Published:** 2019-03-15

**Authors:** Juraj Adamik, G David Roodman, Deborah L Galson

**Affiliations:** ^1^ Department of Medicine Division of Hematology/Oncology, UPMC Hillman Cancer Center, The McGowan Institute for Regenerative Medicine University of Pittsburgh Pittsburgh PA USA; ^2^ Department of Medicine Division of Hematology‐Oncology Indiana University Indianapolis IN USA; ^3^ Richard L Roudebush VA Medical Center Indianapolis IN USA

**Keywords:** MULTIPLE MYELOMA, OSTEOBLASTS, TUMOR‐INDUCED BONE DISEASE, EPIGENETICS

## Abstract

Multiple myeloma (MM) bone disease is characterized by the development of osteolytic lesions, which cause severe complications affecting the morbidity, mortality, and treatment of myeloma patients. Myeloma tumors seeded within the bone microenvironment promote hyperactivation of osteoclasts and suppression of osteoblast differentiation. Because of this prolonged suppression of bone marrow stromal cells’ (BMSCs) differentiation into functioning osteoblasts, bone lesions in patients persist even in the absence of active disease. Current antiresorptive therapy provides insufficient bone anabolic effects to reliably repair MM lesions. It has become widely accepted that myeloma‐exposed BMSCs have an altered phenotype with pro‐inflammatory, immune‐modulatory, anti‐osteogenic, and pro‐adipogenic properties. In this review, we focus on the role of epigenetic‐based modalities in the establishment and maintenance of myeloma‐induced suppression of osteogenic commitment of BMSCs. We will focus on recent studies demonstrating the involvement of chromatin‐modifying enzymes in transcriptional repression of osteogenic genes in MM‐BMSCs. We will further address the epigenetic plasticity in the differentiation commitment of osteoprogenitor cells and assess the involvement of chromatin modifiers in MSC‐lineage switching from osteogenic to adipogenic in the context of the inflammatory myeloma microenvironment. Lastly, we will discuss the potential of employing small molecule epigenetic inhibitors currently used in the MM research as therapeutics and bone anabolic agents in the prevention or repair of osteolytic lesions in MM. © 2019 The Authors. *JBMR Plus* published by Wiley Periodicals, Inc. on behalf of American Society for Bone and Mineral Research.

## Introduction

Patients with multiple myeloma (MM) readily develop osteolytic bone disease that can result in severe bone pain, frequent pathological fractures, and enhanced mortality.^1^ New bone formation at the site of lesions is absent due to MM‐induced suppression of the differentiation of bone marrow stromal cells (BMSCs) into osteoblasts (OBs), enhancing their support of MM growth and drug resistance.^2^, ^3^ New therapies for MM have greatly improved progression‐free survival and overall survival.^4^ However, MM remains incurable for most patients, and MM bone lesions persist even after therapeutic remission of active disease.^5^ It is becoming increasingly evident that multidirectional interactions between MM cells and the surrounding bone microenvironment are the driving factors orchestrating the evolving transformations and heterogenic nature of myeloma tumors.^6^ BMSCs, the multipotent cells with the ability to differentiate into osteoblasts, adipocytes, and chondrocytes,^7^, ^8^ are induced by MM cells to exhibit diverse immune‐modulatory features that are key regulators of myeloma survival and drug resistance and increase support for osteoclast activity.^9^ Direct MM‐BMSC interactions and soluble cytokine‐mediated cross‐talk combine to alter BMSCs into a chronically pro‐adipogenic and senescence‐like phenotype with suppressed osteogenesis.^10^, ^11^, ^12^, ^13^ In fact, the observations that myeloma‐exposed BMSCs (MM‐BMSCs) undergo long‐term phenotypic changes in the absence of myeloma signals suggested that epigenetic modifications direct the cellular reprogramming and osteogenic suppression of MM‐BMSCs.^14^, ^15^, ^16^ In such a way, myeloma‐induced alterations of chromatin structure in BMSCs can be epigenetically propagated as a heritable memory regulating the transcriptional signature in the absence of continuous MM signals. Along with these observations, several analyses comparing the transcriptomic profile of BMSCs cocultured with MM cells and/or patient‐derived MM‐BMSCs to their healthy non‐myeloma counterparts have revealed clear differences.^17^, ^18^, ^19^, ^20^ In this review, we discuss various chromatin‐based epigenetic mechanisms that contribute to osteogenic suppression and enhance adipogenic differentiation of BMSC progenitors in the context of MM bone disease (MMBD). We further address the role for epigenetics in the osteo‐adipogenic switch in the context of normal and malignant bone microenvironments and debate the use of small molecule inhibitors in targeting osteolytic bone destruction in myeloma.

## Epigenetic‐Based Mechanisms and Regulation of Gene Expression

The spatial‐temporal control of gene activation and repression is guided by various epigenetic‐based regulatory systems. Regulation of the epigenome involves various DNA, RNA, and protein‐mediated mechanisms, which define chromatin landscapes and establish the somatic inheritance of cell differentiation states.^21^ Global chromatin histone tail modifications (acetylation, methylation, phosphorylation, and ubiquitination), ATP‐dependent nucleosome remodeling, and DNA methylation patterns define modification‐specific binding domains and transcription factor‐accessible regions that include promoters, enhancers, super‐enhancers, and structural genes to direct lineage‐specific gene expression programs.^22^ Histone‐modifying enzymes can detect and add or remove specific chromatin marks to core histone tails, thus altering the condensation and occupancy of nucleosomes to activate or repress gene transcription.^23^ Mutations in genes encoding chromatin modifiers have been implicated in the vast majority of human cancers including myeloma tumors, which often exhibit remarkable cell heterogeneity encompassing distinct cellular phenotypes.^24^, ^25^

Promoters of many developmental genes are regulated at the level of “bivalent” chromatin signatures, which combine dual activation‐specific histone H3 lysine 4 trimethylation (H3K4me3) and repressive H3K27me3 modifications. These gene promoters are “poised” for transcriptional activation or repression in a spatial‐temporal and cell‐specific differentiation pattern.^26^, ^27^ Bernhart and colleagues^28^ reported that in contrast to normal cells, the DNA of bivalent promoters are often hypermethylated in cancer samples, unexpectedly leading to overexpression of developmental growth‐promoting factors and cancer‐associated genes in fresh cancer tissues. Malignant plasma cells from MM patients have distinct genomic profiles of H3K27me3, H3K4me3, and bivalent promoter modifications that differ from normal donor plasma cells. Global increases in the number of bivalent and H3K27me3‐modified genes in patient‐derived MM cells correlated with advanced stages of disease and poor survival.^29^ Polycomb‐group proteins are a family of transcriptional repressor proteins, which regulate the deposition and maintenance of H3K27me3 repressed chromatin domains that play roles in MM tumorigenesis.^30^ The two major polycomb repressive complexes are PRC1 and PRC2. PRC1 complexes have E3 ligase activity and contribute to the methylation of H3K27 by catalyzing the mono‐ubiquitination of histone H2A at lysine 119 (H2AK119). PRC2 is a trimeric complex consisting of SUZ12, EED, and methyltransferase subunits Enhancer of Zeste Homolog 2/1 (EZH2/EZH1), which catalyze di‐ and trimethylation of H3K27 (H3K27me2/3).^30^ Both PRC1/2 core components can cooperate with additional chromatin modifiers, non‐coding RNAs, and transcription factors that regulate their enzymatic activity and/or define the mode of their recruitment to target genes to regulate the stem cell–like features of cells and their growth and differentiation.^31^, ^32^, ^33^ The repressive activity of EZH2 is countered by proteins tetratri‐copeptide repeat X chromosome (UTX/KDM6A) and jumonji domain containing 3 (JMJD3/KDM6B). These histone lysine demethylases remove methyl groups from H3K27me3 to promote gene expression during cell differentiation.^34^

## Chromatin Alterations in MM‐Exposed BMSCs

**Figure 1:**
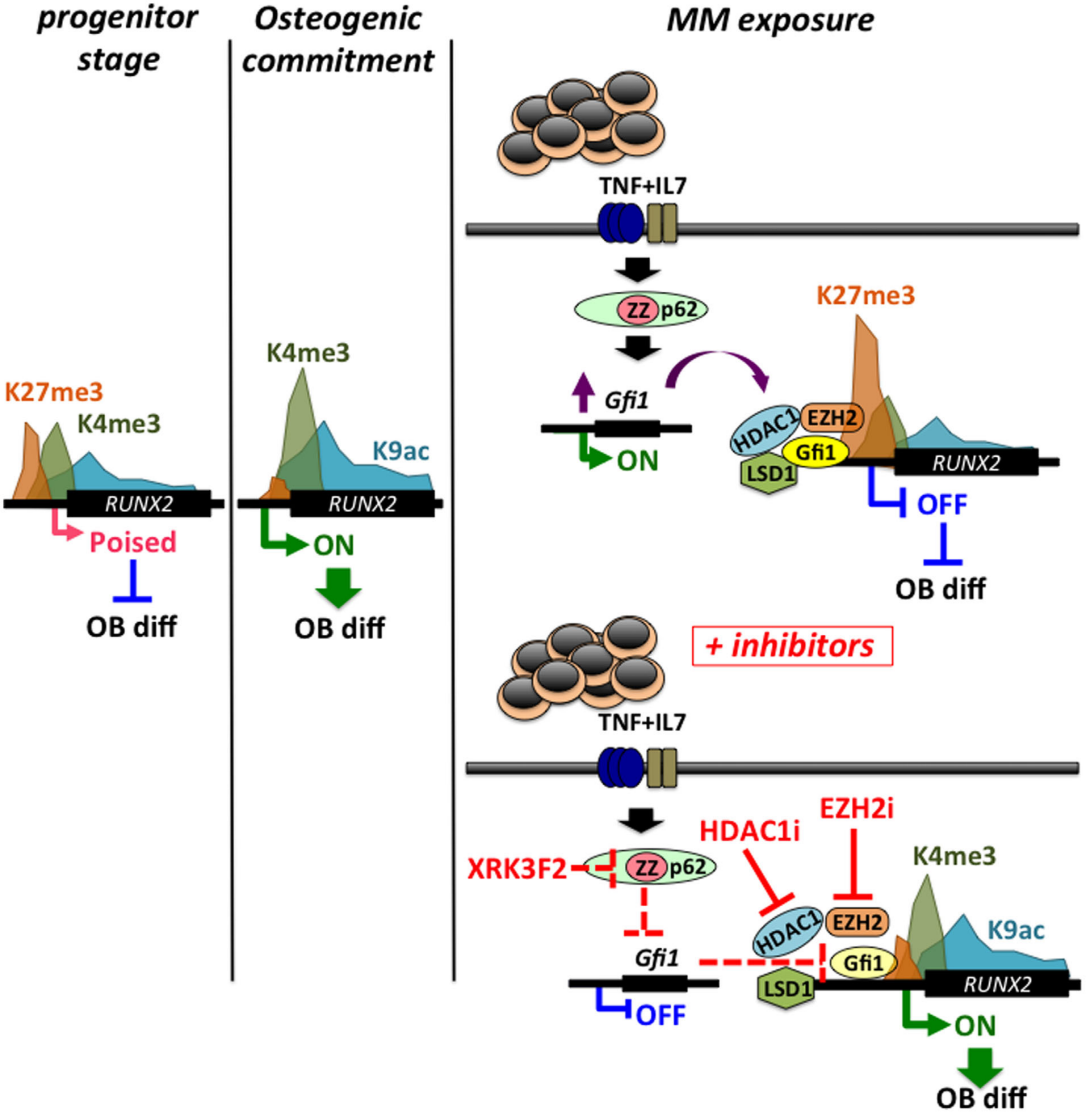
Chromatin suppression of *RUNX2* promoter in MM‐BMSCs. In undifferentiated BMSCs, *RUNX2* is in a transcriptionally permissive state with bivalent promoter architecture having active H3K4me3 and repressive H3K27me3 chromatin marks. During OB differentiation, the *RUNX2* promoter has elevated H3K4me3 and H3K9ac and decreased H3K27me3 levels denoting an open/euchromatic and transcriptionally active state. MM exposure induces binding of the transcriptional repressor Gfi1 to the *RUNX2* promoter, resulting in recruitment of chromatin modifiers EZH2, HDAC1, and LSD1. These modifiers deposit repressive chromatin marks on *RUNX2* promoter core histones and epigenetically block its transcription. The active chromatin signature of *RUNX2* changes into a repressive H3K27me3‐prevalent state. The use of small molecule inhibitors targeting HDAC1 and EZH2 reverses the inhibitory chromatin effects and enhances *RUNX2* transcription. In addition, blocking p62‐mediated activation of Gfi1 and its binding to the *RUNX2* promoter using the ZZ‐domain inhibitor XRK3F2 prevents HDAC1 recruitment and reactivates *RUXN2* expression.

A recent study by Adamik and colleagues^15^ demonstrated that inhibition of osteogenesis in myeloma cocultured and/or patient‐derived MM‐BMSCs is largely due to heterochromatin silencing of the promoter of the key osteogenic factor *RUNX2*.^35^ *RUNX2/CBFA1* is required for OB differentiation, and its expression is reduced in osteoprogenitors from bone marrow biopsies of MM patients with osteolytic lesions.^36^ In contrast, its elevated expression in MM cells has been shown to promote MM tumor growth and associated bone disease.^37^ Epigenetic‐based mechanism studies in MM‐BMSCs followed the work by D'Souza and colleagues,^38^ which revealed the role for the transcription factor growth factor independence‐1 (Gfi1) in repression of *RUNX2* gene expression. Gfi1 is a SNAG (Snail/Gfi1) domain‐containing C_2_H_2_ zinc‐finger involved in differentiation of lymphoid and myeloid cells^39^ and new research suggests its deregulation in various hematologic malignancies including myeloma.^40^, ^41^, ^42^, ^43^ BMSCs exposed to MM cocultures or harvested from either a murine MM model or MM patients have increased Gfi1 expression. Further, BMSC from Gfi1‐knockout mice or Gfi1 knockdown in murine OB precursors (pre‐OBs) before MM exposure significantly protected the cells from MM suppression with improved response to OB differentiation signals.^16^, ^38^ Importantly, knockdown of Gfi1 after MM exposure of murine pre‐OB or in patient‐derived MM‐BMSCs could reverse the OB suppression and enhanced response to OB differentiation signals. Transcriptional repression by Gfi1 is dependent on its recruitment of histone‐modifying enzymes histone deacetylase 1 (HDAC1), lysine‐specific histone demethylase 1 (LSD1/KDM1A), methyltransferase G9a, and EZH2 to target gene promoters.^15^, ^16^, ^38^, ^44^, ^45^ The first evidence of Gfi1‐mediated chromatin suppression of *RUNX2* in the realm of myeloma suppression came from an experiment showing that overexpression of Gfi1 in preOBs inhibited *RUNX2* reporter expression, and this was prevented by treatment with the HDAC inhibitor Trichostatin A.^38^ Further studies characterized Gfi1 binding sites within the *RUNX2* promoter and demonstrated that after MM exposure, Gfi1 recruits EZH2, HDAC1, and LSD1 to alter the bivalent signature of the *RUNX2* promoter into one predominantly methylated at H3K27me3^15^ (Fig. 1). This repressed heterochromatic state at the *RUNX2* promoter persisted for several days after removal of MM cells from the cocultures and was refractory to OB differentiation signals. The use of small molecule inhibitors targeting HDAC1 or EZH2 activity rescued expression of *RUNX2* with its downstream targets and enhanced osteogenic differentiation of MM‐pretreated murine MC3T3‐E1 preOB cells and patient‐derived MM‐BMSCs^15^ (Fig. 1). In a subsequent study, a novel small molecule inhibitor of signaling via the ZZ domain of p62 (Sequestosome 1), XRK3F2, blocked tumor necrosis factor (TNF) and multiple myeloma‐induced Gfi1 upregulation, resulting in decreased binding and recruitment of HDAC1 to the *RUNX2* promoter in pre‐OBs.^16^ These results complement previous in vivo observations in the intratibial‐injected 5TGM1 MM‐KaLwRij syngeneic murine model of MMBD, in which XRK3F2 induced new cortical bone formation in MM‐injected limbs.^46^ Collectively, these data argue for the importance of the p62‐ZZ‐domain‐Gfi1 axis in converging the extracellular myeloma signals to HDAC1/EZH2‐mediated epigenetic gene silencing in MM‐BMSC. In addition to *RUNX2*, *Osteopontin* (*OPN*) was also shown to be a Gfi1‐regulated gene in a study by Wang and colleagues.^47^ They showed that AMP‐activated protein kinase (AMPK) signaling promoted osteogenesis by downregulating Gfi1 and derepressing *OPN* expression, which resulted in enhanced ectopic bone formation from AMPKα transduced MC3T3‐E1 pre‐OBs placed into nude mice.^47^ Bioinformatics analyses by Garcia‐Gomez and colleagues^19^ suggested that putative Gfi1 binding sites are among the highest represented transcription factor binding sites located in the promoters of deregulated genes in MM cocultured BMSCs. Therefore, it would be informative to conduct a genomewide Gfi1 chromatin immune precipitation (ChIP) analyses coupled with total RNA sequencing to define the spatial‐temporal nature of the myeloma‐inducible Gfi1 regulatory cistrome in MM‐BMSCs.

## Impact of Obesity and High‐Fat Diet on MMBD

Aging, obesity, chronic drug treatments, and various pathological inflammatory disease states, including MM, are collectively associated with a decrease in bone mass and a concomitant increase in bone marrow adiposity.^48^, ^49^ Studies in vivo suggest that changes in the bone microenvironment that occur with aging are the major factors driving adipogenic differentiation of mesenchymal progenitors.^50^ This age‐dependent increase in marrow adipocytes is closely associated with decreased bone function and hematopoiesis and correlates with osteoporosis and increased fracture risk.^51^ The conventional role for adipose tissue in energy homeostasis is becoming extensively revised, and new evidence reviewed extensively by Bukowska and colleagues^52^ points out that adipose tissue plays a significant metabolic and endocrine role in body physiology and disease development. White adipose tissue and brown adipose tissue are the two major types of fat tissues. Although the more predominant white adipose tissue partakes in energy storage, insulation, mechanical support for internal organs, and endocrine and immune modulation, brown adipocytes have extensive thermogenic properties.^52^ Bone marrow adipose tissue residing on the endosteal surface and throughout the bone marrow cavities has a complex set of regulatory functions directly impacting the osteogenic, angiogenic, and immunogenic properties of the bone marrow niche.^53^ Increased marrow adiposity can have multiple effects on the marrow and bone because adipocytes express and secrete multiple factors, such as adepsin, leptin, adiponectin, TNF, and angiotensinogen with significant pleiotropic effects on the local microenvironment of bone marrow cells.

Obesity is a significant risk factor associated with development of MM and increased morbidity of MM patients.^54^, ^55^ High‐fat diet–induced obesity in mice increases the likelihood for development of monoclonal gammopathy of undetermined significance (MGUS)‐like disease with increased MM cell accumulation and significant bone loss.^56^ Trotter and colleagues^57^ demonstrated that bone marrows from MM patients contain increased numbers of pre‐adipocytes and mature adipocytes, which secrete multiple factors including MIP1 and SDF1α that support MM cell growth, chemotaxis, and increased tumor growth in bones in vivo. Further, adipocytes from obese patients displayed deregulated hormonal and signaling that enhance MM growth, adherence, and their angiogenic potential.^58^

Recent studies have provided a mechanistic basis for the clinical observation that those patients with a greater degree of bone destruction present with higher adipocyte numbers when compared with patients with milder osteolytic bone involvement.^53^ Yang and colleagues reported that coculture of BMSCs with MM cells enhanced adipocyte differentiation in an integrin α4β1‐dependent manner in vitro and increased adipocyte content in the bone marrows when MM cells were injected into human or murine bones.^59^ The osteocyte‐derived Wnt‐inhibitor Sclerostin (SOST), implicated in the anti‐osteogenic effects of MM,^60^ was recently shown to enhance adipocyte differentiation of murine and human BMSCs.^61^ MM cells with elevated levels of heparanase, an enzyme that cleaves heparin sulfate chains of proteoglycans and has been linked to uncoupled bone destruction in MM,^62^ significantly suppressed bone formation in a mouse MM model in vivo compared with MM cells with low heparanase.^63^ Similarly, elevated heparanase in MM conditioned media inhibited mineralization by OBs in vitro.^63^ Further, heparanase enhanced pre‐OB and MM cell expression and secretion of receptor activator of nuclear factor kappa‐B ligand and the Wnt pathway inhibitor Dickkopf‐1, which increased osteoclastogenesis and promoted adipogenic differentiation of OB progenitors, respectively.^63^ These studies suggest that the pro‐inflammatory MM bone marrow environment shifts differentiation of BMSCs toward adipogenesis to enhance tumor cell growth.

## Epigenetic Contributions to the Osteo‐Adipogenic Switch of BMSCs in MMBD

Osteoblasts and adipocytes are derived from a common mesenchymal lineage precursor. Whether BMSCs undergo osteogenic or adipogenic differentiation is dependent on the activation of phenotype‐specific transcription factors and coordinated epigenetic reprograming of specific genes that provide precise spatial and temporal control of gene expression. Meyer and colleagues^64^ showed that the epigenetic profiles of undifferentiated BMSCs closely resembled those of predifferentiated osteogenic cells and that a greater number of genetic changes is required for adipogenic compared with osteogenic differentiation. After exposure of primary human BMSCs to lineage‐specific differentiation factors, the genetic and epigenetic reprogramming that drives BMSC differentiation to OB or adipocytes is initiated within the first 3 hours and established by the first 2 days.^65^ Although OB differentiation appears to be the default pathway for BMSCs’ differentiation, this preprogramed epigenetic differentiation property of BMSCs can be subverted by pathological conditions that suppress osteogenesis and increase adipogenesis.^64^

**Figure 2:**
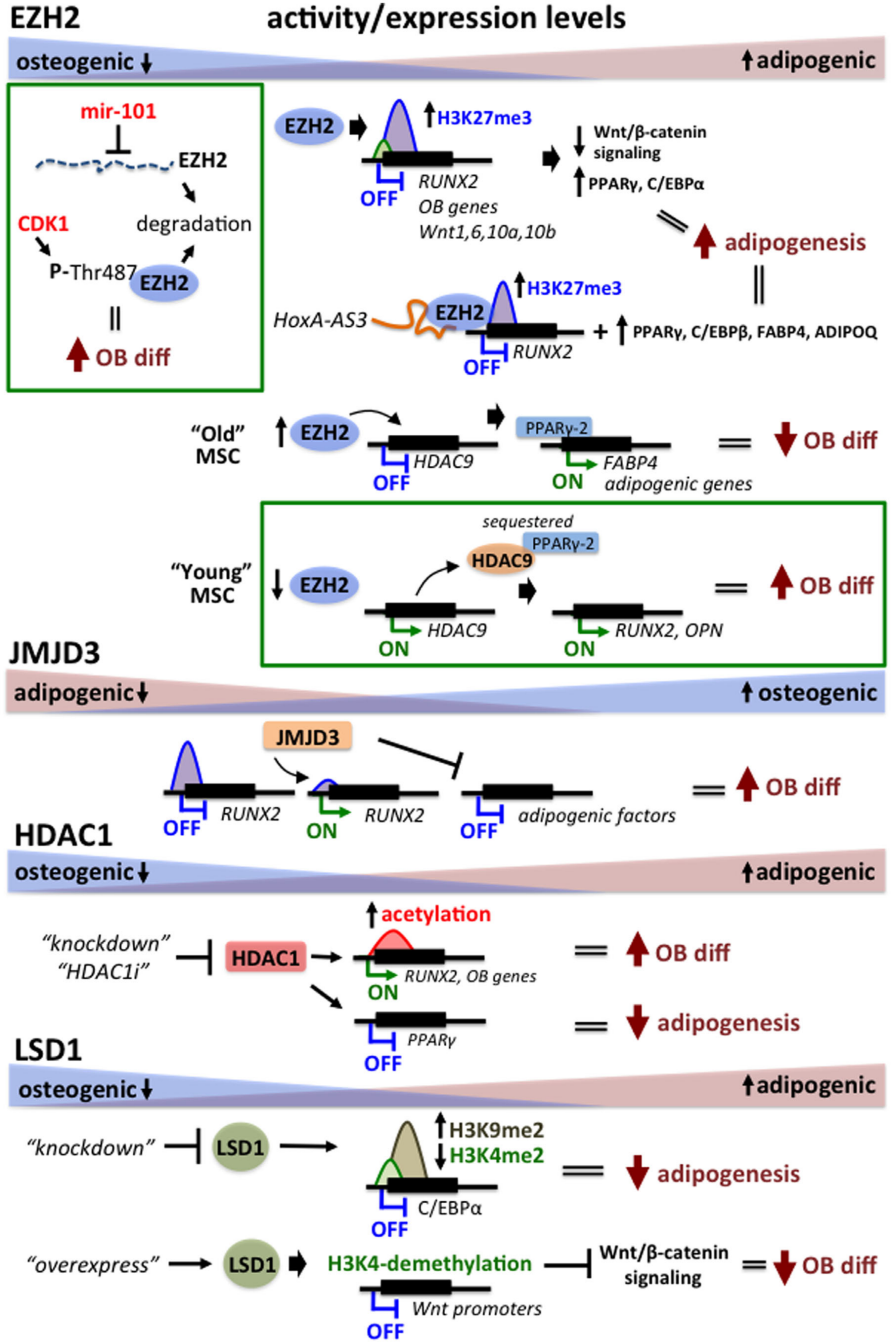
Expression and action of histone modifiers during osteogenic and adipogenic differentiation. EZH2 is downregulated in differentiating OBs by a variety of mechanisms, including miRNA‐targeted and phosphorylation‐mediated degradation. EZH2 blocks osteogenesis and promotes adipogenic differentiation by H3K27me3‐mediated suppression of several classes of osteogenic genes, including *RUNX2* and bone stimulatory Wnt genes Wnt1, −6, −10a, and −10b. By blocking Wnt/β‐catenin signaling, EZH2 permits expression of the key adipogenic factors peroxisome PPARγ and C/EBPα. EZH2 can complex with *HoxA‐AS3* to repress *RUNX2* during adipogenic differentiation. In addition, elevated *HoxA‐AS3* was shown to up regulate expression of adipogenic markers PPARγ, C/EBPα, FABP4, and ADIPOQ in mesenchymal stem cell precursors. EZH2 has been implicated in HDAC9c‐PPARγ regulation of age‐dependent osteogenic and adipogenic differentiation of BMSCs. EZH2 expression increases with aging and represses expression of HDAC9, which allows PPARγ to activate adipogenic gene expression. In young BMSCs EZH2 expression is low, allowing for HDAC9 expression and sequestration of PPARγ to prevent activation of adipogenesis and enhance expression of osteogenic genes *RUNX2* and *OPN*. Overexpression of JMJD3 during OB differentiation positively regulates transcriptional activity of *RUNX2* by counteracting repressive H3K27me3 chromatin mark and decreases expression of adipogenic transcription factors. HDAC1 enzymatic activity and expression declines during OB differentiation and inhibition of HDAC1 in BMSCs activated *RUNX2* and decreased expression of PPARγ, which favored osteogenic differentiation and reduced lipid accumulation and blocked adipogenic differentiation. LSD1 activity increases during adipogenesis, and its knockdown promotes osteogenic differentiation due to decrease in activation‐specific H3K4me2 and increase in repressive H3K9me2 mark at the promoter of the adipogenic transcription factor C/EBPα. Overexpression of LSD1 in MSCs induced H3K4 demethylation and epigenetic silencing of pro‐osteogenic Wnt‐gene promoters, which blocked OB differentiation.

The chromatin‐based mechanisms responsible for the pathologic switch in BMSC differentiation toward adipocytes in MMBD are largely unknown. One possibility is that upregulation of Gfi1 and its co‐repressors HDAC1, LSD1, and EZH2 in MM‐BMSCs may have more widespread epigenetic effects beyond regulation of the *RUNX2* gene. These factors may both suppress and shift the osteogenic potential of MM‐BMSCs toward adipogenesis. Recent studies reported that in addition to being a potent transcriptional suppressor of osteogenic differentiation,^14^, ^15^ Gfi1 plays a role downstream of AMPKα in regulating adipogenesis.^47^ Overexpression of wild‐type Gfi1 increased adipogenesis and intracellular fat droplet content of AMPKα activated 3T3‐L1 cells.^47^ Further, in agreement with its role in MM‐induced suppression of OB differentiation, EZH2 is a well‐accepted negative regulator of osteogenesis.^66^ EZH2 plays a critical role during neural‐crest cell‐derived cartilage differentiation, osteogenic differentiation, and skeletal patterning during development^67^, ^68^, ^69^, ^70^, ^71^, ^72^ (for review, see Dudakovic and van Wijnen^66^). EZH2 is subjected to a variety of posttranscriptional (eg, miR‐101‐mediated)^73^ and posttranslational (eg, CDK1‐phosphorylation at Thr487)^74^ regulatory mechanisms that ensure its degradation and downregulation during osteogenic commitment of BMSCs (Fig. 2). EZH2 blocks osteogenesis, in part, via generation of H3K27me3 suppression of several classes of osteogenic gene promoters, including *RUNX2*.^70^, ^75^, ^76^, ^77^ Further, EZH2 also inhibits the Wnt/β‐catenin signaling pathway by directly targeting bone stimulatory Wnt genes Wnt1, −6, −10a, and −10b, to promote adipogenic differentiation of mouse peripheral preadipocytes and primary mesenchymal stem cells.^78^ By blocking Wnt/β‐catenin signaling, EZH2 permits expression of peroxisome proliferator‐activated receptor‐γ (PPARγ) and CCAAT/enhancer binding protein α (C/EBPα), which are the principal adipogenic transcription factors (Fig. 2).^79^ Interestingly, the EZH2‐HDAC9c‐axis has also been recently implicated in age‐dependent osteogenic and adipogenic differentiation of BMSCs.^80^ These experiments demonstrated that EZH2 expression increased with aging of primary mouse and human BMSCs, thereby increasing expression of PPARγ and allowing progression of adipogenesis. Inhibition of EZH2 reduced adipogenic differentiation of aged BMSCs and rescued their capacity for osteogenic differentiation.^80^ Jing and colleagues^81^ reported that elevated EZH2 expression in BMSCs correlated with increased H3K27me3 levels on the Wnt1, Wnt6, and Wnt10a promoters and resulted in their commitment to adipocyte differentiation with osteoporosis. Restoring canonical Wnt signaling by knockdown of EZH2 and the use of EZH2 inhibitor 3‐deazaneplanocin A (DZNep) prevented the shift of BMSCs into adipocytes and enhanced their osteogenic differentiation.^81^ A recent report demonstrated that interaction between EZH2 and long non‐coding RNA *HoxA‐AS3* is involved in repressing *RUNX2* during adipogenic differentiation. In addition, silencing of *HoxA‐AS3* in BMSC precursors decreased expression of adipogenic markers PPARγ, C/EBPα, FABP4, and ADIPOQ and resulted in inhibition of adipogenesis (Fig. 2).^82^ In MMBD, MM cell exposure of OB progenitors stimulated recruitment of EZH2 to RUNX2 and epigenetic repression of *RUNX2* transcription, thereby suppressing osteogenesis.^15^ Further, the selective EZH2 inhibitor GSK126 rescued *RUNX2* expression as well as expression of its downstream osteogenic‐target genes and enhanced osteogenic differentiation of MM‐exposed pre‐OBs as well as MM‐patient‐derived BMSCs.^15^ Since BMSCs isolated from MM‐injected tibias of mice and primary MM patient marrows still retain their capacity to differentiate into adipocytes,^38^ given the experimental evidence of EZH2's involvement in regulating the adipogenetic switch, these results suggest that EZH2 is likely involved in the epigenetic regulation of the osteo‐adipogenic imbalance in MM‐involved bone.

EZH2 and JMJD3 exhibit opposing functions during BMSC differentiation.^76^ Homozygous deletion of JMJD3 delayed OB differentiation and bone formation in mice.^83^ Overexpression of JMJD3 decreased expression of adipogenic transcription factors and positively regulated transcriptional activity of *RUNX2* (Fig. 2).^84^ Although regulation of JMJD3 in BMSCs is not well understood, miR‐146a can target JMJD3 expression and prevent OB differentiation.^85^ In a similar fashion, *UTX*‐deficient preadipocytes and BMSCs exhibit enhanced adipogenesis and decreased osteogenesis due to their deregulated Wnt/β‐catenin/c‐Myc pathways.^86^ Interestingly, we found that JMJD3 is downregulated in MM‐BMSCs, which prevents the derepression of heterochromatin at *RUNX2* and subsequent suppression of OB differentiation (Fig. 2) (unpublished data presented at ASBMR, 2016).

The role of HDACs in osteogenic differentiation and bone development has been extensively characterized. Collectively, these deacetylases regulate numerous cellular events, including gene transcription, cytoskeletal dynamics, and a plethora of signaling pathways during development and aging.^87^ HDAC1 enzymatic activity and expression declines during OB differentiation, and knockdown or small molecule inhibition of HDAC1 in BMSC progenitors stimulates osteogenic gene expression and OB differentiation.^88^ Further, pharmacological inhibition and genetic deletion of *HDAC1* in cultured mesenchymal precursor cells caused reduced lipid accumulation and blocked adipogenic differentiation of these cells.^89^ In support of this, HDAC1 knockdown in hASCs resulted in acetylation and activation of *RUNX2* together with decreased expression of PPARγ, which favored osteogenic differentiation (Fig. 2).^90^

LSD1 has also been identified as a key epigenetic regulator in brown adipogenesis, and inhibition or depletion of LSD1 repressed brown adipocyte tissue differentiation in vitro and in vivo.^91^ Knockdown of LSD1 decreased differentiation of 3T3‐L1 preadipocytes. The impaired adipocyte differentiation was associated with decreased transcriptionally permissive H3K4 dimethylation and increased repressive H3K9 dimethylation at the promoter of the adipogenic transcription factor C/EBPα (Fig. 2).^92^ Similarly, increased levels of LSD1 repressed osteogenic differentiation and promoted brown adipogenesis. Overexpression of LSD1 in BMSCs blocked Wnt signaling pathway by demethylating H3K4 and epigenetically silencing pro‐osteogenic Wnt‐gene promoters (Fig. 2).^91^ In addition, knockdown of LSD1 or its partner REST corepressor 2 (rcor2) blocked adipogenesis and increased expression of inflammatory cytokines and chemokines in 3T3‐L1 preadipocytes.^93^ Collectively, these results suggest that the use of small molecule inhibitors to epigenetically target EZH2, HDAC1, and LSD1 could reverse adipogenic differentiation that occurs at the expense of suppressed osteogenesis and promote bone repair in MM.

## Epigenetic Targeting as Treatment of MMBD

There are several classes of inhibitors that target different aspects of epigenetic pathways. However, the main challenge associated with the use of epigenetic inhibitors is their broad effects and lack of cellular specificity. Despite this, to date several classes and types of epigenetic inhibitors, which primarily target molecular complexes and catalytic domains of chromatin‐modifying enzymes, are in preclinical trials and/or have been approved for cancer treatment. However, our understanding of the epigenetic‐based mechanisms in the anabolic and catabolic responses that control bone homeostasis in bone pathologies like MM is currently limited and the epigenetic basis of MM‐induced osteogenic suppression of BMSCs in the presence of bone destruction is largely understudied. Thus, there is a great need and requirement to determine the effects of epigenetic inhibitors on normal bone physiology and tumor‐associated bone disease. Tables 1 and 2 summarize the effects of several classes of current antimyeloma drugs and epigenetic inhibitors described in later sections.

**Table 1:** Anticancer Drugs Used in Combination With Epigenetic Inhibitors

Target/inhibitor	Action/response	Reference no.
Bortezomib Carfilzomib	Bortezomib is a reversible (boronic acid‐based) and carfilzomib is a irreversible (epoxyketone‐based) proteasome inhibitor. By targeting the ubiquitin‐proteasome pathway system, which regulates protein degradation, these agents effectively interfere with cell cycle control, angiogenesis, and induce apoptosis of cancer cells.	101
Lenalidomide Pomalidomide	These compounds are derivatives of thalidomide, which is a teratogen and potent inhibitor of angiogenesis. Both of these immunomodulatory drugs exhibit direct antitumor effects with anti‐angiogenic and anti‐inflammatory properties.	102
Melphalan	Melphalan is a derivative of chlormethine. This alkylating agent induces DNA adducts, which results in DNA interstrand cross‐linking with cytotoxic effects against cancer cells.	113

**Table 2:** Epigenetic Inhibitors and Their Action on MM Survival and Osteoblastogenesis

Target/Inhibitor	Action/Response	Reference
DNMTi	anti‐MM/pro‐osteogenic	
azacitidine	anti‐MM effects (inducing cell cycle arrest)	94
	induced proliferation, ALPL activity and mineralization of older donor hASCs	96
	inhibited DNA methylation in 3T3‐L1 preadipocytes, inhibited adipogenesis, promoted osteoblastogenesis and re‐expression of *WNT10A*	97
decitabine	in combination with HDACi quisinostat in vivo 5T33MM ‐ blocked MM cell growth	95
HDACi	anti‐MM/mixed effects on OB diff; high/low dose ‐ anti/pro‐osteogenic respectively	
vorinostat + SAHA	MM cell apoptosis due to changes in genes guiding cytokine‐driven proliferation and survival, drug‐resistance, cell cycle control, DNA synthesis/repair, and proteasome function	100
panobinostat	in combination therapies with bortezomib and IMiDs exhibits anti‐MM effects	102
SAHA	induced cell cycle arrest, DNA damage and decreased osteogenic gene expression resulted in suppressed osteogenic colony formation by BMSCs	103
vorinostat	inhibited boone tumor burden in pre‐clinical models of bone metastatic breast (MDA‐231) and prostate (PC3) cancers, compromised the normal trabecular bone mass in mice 4 weeks of therapy caused significant osteopenia in the contralateral non–tumor‐bearing femurs and limbs from tumor‐free SCID mice	104 107,108
	less frequent and optimized treatment regimen *in vivo*, and the use of lower‐doses for *in vitro* cell treatments promoted osteogenic differentiation by ND‐BMSCs increased ALPL activity, mRNA of osteogenic markers and Ca2+ deposition in patient MM‐BMSCs	
valproic acid (VPA) sodium butyrate (NaB) trichostatin A (TSA)	treatment of adipose and umbilical cord MSC increased and favored osteogenic differentiation over adipogenic, chondrogenic, and neurogenic differentiation	105, 106
	enhanced expression of RUNX2 and osteogenic genes, and exhibited concentration dependent positive effects on OB maturation *in vitro* and *in ex vivo* calvarial organ cultures.	107
	low dose treatment increased cell proliferation, enhanced mineralized nodule formation by preOBs, higher concentrations exhibited cytotoxic effects	
JNJ‐26481585 (low dose)	in combo of bortezomib reduced OCLs and increased OBs, trabecular bone volume, and trabecular number as compared to bortezomib alone	109
HDAC1i MC‐1294	rescued expression of osteogenic genes *ALPL, RUNX2, OCN, BSP* in MM exposed preOBs and enhanced mineralization of patient MM‐BMMSCs	15
BET/BRD	anti‐MM effects/anti‐osteogenic effects	
JQ1	potent anti‐MM effects *in vitro/in vivo* (cell cycle arrest and senescence)	111
	induces cell growth arrest and caspase‐mediated apoptosis by downregulation of *c‐MYC* and its target genes in MM	111, 112
	effective against primary osteosarcoma tumors in vivo, decreased OB differentiation (targets *MYC* and *RUNX2* expression)	118
I‐BET151 JQ1	suppressed chondrocyte differentiation *in vitro* and reduced bone growth *in vivo* in a zebrafish model	116
BRDi CPI203	effective against melphalan and bortezomib resistant MM cells *in vitro*	114
	synergized with bortezomib and melphalan as well as lenalidomide and dexamethasone to induce MM cell apoptosis	114, 115
	prevented BMSC‐mediated protection of MM cells	115

**EZH2i**	**anti‐MM effects/osteo‐protective effects**	
tazemetostat (EPZ‐6438) GSK2816126 CPI‐1205 = (GSK126)	phase I/II clinical trials in patients with diffuse large B‐cell and follicular lymphomas and genetically defined solid tumors	119
UNC1999, GSK343, GSK126 DZNep EPZ005687	Inhibition of EZH2 induced apoptosis in a variety of MM cell lines	29, 128, 129, 131, 132
E7438	reduced subcutaneous growth of MM cell xenographs in mice	130
EPZ‐6438	in combo with azacitidine resensitized IMiD‐resistant OPM2 and NCI‐H929 MM cells to lenalidomide and pomalidomide	133
DZNep	*in vivo* administration enhanced osteogenic differentiation of BMSCs	81
GSK126	inhibited proliferation of MM cells with stem cell‐like characteristics	132
	increased bone density in wild type adult mice and estrogen‐deficient (OVX) mice	66,69,136
	rescued osteogenesis of MM‐exposed preOBs and patient BMSCs	15
	synergized with bortezomib with anti‐MM effects in 3D MM cultures	137
dual EZH2/1i UNC1999	sensitized MM cells to proteasome inhibitors	134
dual EZH2/1i OR‐S1	eradicated minimal residual disease from the bone marrow in an orthopic MM model, reduced IgG serum levels in a MM patient‐derived xenograft mouse model	135
BMI1i	anti‐MM/adverse effects on skeletal progenitors	
PTC209	induced apoptosis in MM cell lines and primary MM cells *in vitro*	140
	synergistic and additive anti‐MM effects when combined with pomalidomide and carfilzomib	142
	anti‐MM effects in the presence of IGF1, IL6 and in cocultures with of BMSCs	
	impaired OB formation in a dose‐dependent manner due to elevated expression of DKK	
	synergistic and additive anti‐MM effects with EZH2i and BETi	129
BMI1 shRNA	effective in reducing tumor growth of MM xenografts in mice	140
overexpression of BMI1	enhanced osteogenic differentiation of hASCs and increased BMP2 and WNT11	145
knockdown of BMI1	upregulated PPARγ and blocked osteogenesis of human embryonic and induced pluripotent MSCs	142
BMI1 KO mice	skeletal growth retardation, decreased trabecular bone volume and bone mineral density	146
	exhaustion of the BMSCs pool, impaired OB differentiation markers and mineral deposition with increased PPARγ and the number of bone marrow adipocytes	
LSD1i	anti‐MM effects/inhibits osteogenesis and supports adipogenesis	
knockdown of LSD1	enhanced the cytotoxicity of HDACi (SAHA, LBH589), reduced expression of surface adhesion proteins and migration/ invasion of MM1.S cells	148
	increased density and ossification of hASCs as compared to scrambled controls	152
plant extract, triptolide	induced cell‐cycle arrest and apoptosis of RPMI8226 cells	150
pargyline, CBB1007	have been shown to enhance ALPL activity and ECM mineralization by hASCs enhanced H3K4me2 at the promoters of osteogenic genes, rescued OB differentiation	151,152
pargyline	restored the osteogensis of *ex‐vivo* expanded BMSCs from aged osteoporotic and/or OVX mice	151

DNA methyltransferase inhibitors (DNMTi), eg, azacitidine and decitabine, have been reported to have anti‐MM effects by inducing cell cycle arrest and impacting the growth of resistant MM cell lines and primary patient‐derived MM cells.^94^ Maes and colleagues^95^ used a combination of the DNMTi decitabine and the HDACi quisinostat in the in vivo syngeneic 5T33MM mouse model to test the antitumor activity of these epigenetic modulating agents. They showed that the agents induced transcriptional responses in MM cells that blocked MM cell growth. Azacitidine treatment induced proliferation and increased alkaline phosphatase activity and matrix mineralization by adipose‐derived mesenchymal stem cells isolated from older donor patients who had impaired osteogenic potential.^96^ Azacytidine‐induced inhibition of DNA methylation in 3T3‐L1 preadipocytes significantly inhibited adipogenesis and promoted osteoblastogenesis that induced re‐expression of *WNT10A*.^97^

Similarly, histone deacetylase inhibitors (HDACi), including vorinostat and panobinostat, are being used to treat a wide range of hematologic malignancies.^98^ These small‐molecule inhibitors have a broad range of antitumor effects, including cell cycle arrest, induction of apoptosis, cell differentiation, autophagy, and anti‐angiogenic effects on cancer cells.^99^ Treatment with vorinostat/suberoylanilide hydroxamic acid (SAHA) caused MM cell apoptosis due to profound changes in expression of genes mediating cytokine‐driven proliferation and survival, drug resistance, cell cycle control, DNA synthesis/repair, and proteasome function.^100^ The FDA‐approved HDAC, panobinostat, has been used effectively in combination therapies with the anti‐MM agents bortezomib^101^ and immunomodulatory drugs (IMiDs).^102^ The effects of pan‐HDACs on bone formation are not completely understood and so far have had mixed results. McGee‐Lawrence and colleagues^103^ reported that SAHA induced bone loss with a reduction in OB numbers in vivo.^103^ In vitro, SAHA treatment induced cell cycle arrest, DNA damage, and decreased osteogenic gene expression that resulted in suppressed osteogenic colony formation by isolated BMSCs.^103^ In preclinical models of bone metastatic breast (MDA‐231) and prostate (PC3) cancers, the pan‐HDACi vorinostat effectively inhibited tumor burden in bone but also had a negative systemic effect and compromised the normal trabecular bone mass in mice. After 4 weeks of therapy, the contralateral non‐tumor‐bearing femurs and limbs from vorinostat‐treated tumor‐free SCID mice showed significant osteopenia.^104^ In contrast, treatment of adipose and umbilical cord mesenchymal stem cells with HDAC inhibitors valproic acid, sodium butyrate, and trichostatin A^105^, ^106^ increased and favored osteogenic differentiation over adipogenic, chondrogenic, and neurogenic differentiation. Schroeder and colleagues^107^ showed that HDACi enhanced expression of *RUNX2* and osteogenic genes and exhibited concentration‐dependent positive effects on OB maturation in vitro and in ex vivo calvarial organ cultures. Low‐dose valproic acid, sodium butyrate, and trichostatin A treatment increased cell proliferation and enhanced mineralized nodule formation by pre‐OBs, although higher concentrations of HDACi exhibited considerable cytotoxic effects.^107^ Additional studies demonstrated that using a less frequent and optimized vorinostat treatment regimen in vivo and lower doses of vorinostat for in vitro cell treatments promoted osteogenic differentiation by healthy donor BMSCs.^108^ Further, this study showed that vorinostat treatment increased alkaline phosphatase activity, mRNA expression of osteogenic markers, and calcium deposition in patient‐derived MM‐BMSCs.^108^ More importantly, combinations of bortezomib with low doses of the HDACi JNJ‐26481585 induced a more pronounced reduction of osteoclasts and increased OBs, trabecular bone volume, and trabecular number when compared with bortezomib used as a single agent.^109^ Inhibition of HDAC1 with the selective inhibitor MC‐1294 rescued expression of osteogenic genes *ALPL*, *RUNX2*, *OCN*, and *BSP* in MM‐exposed pre‐OBs and enhanced mineralization of patient‐derived MM‐BMSCs.^15^ Because the major challenge in the use of pan‐HDAC inhibitors is their lack of specificity, targeting specific members of the HDAC family of proteins may be more beneficial with lessened side effects for treating bone metastatic cancers.

The bromodomain and extra‐terminal domain (BET) motif‐containing family of proteins binds acetylated lysines on histone tails and recruits histone‐modifying enzymes to regulate chromatin structure and gene expression. The bromodomain family consists of four proteins (BRD2, BRD3, BRD4, and BRDT), and compelling preclinical data demonstrate that BET domain targeting may be a valuable strategy for treating a wide range of solid tumors and hematologic malignancies.^110^ The BET inhibitor JQ1 exhibited potent antiproliferative effects associated with cell cycle arrest and cellular senescence of MM cell lines and primary CD138+ patient‐derived MM cells and significantly decreased MM tumor burden in vivo.^111^ The anti‐MM effects of BET inhibitors result from their inducing cell growth arrest and caspase‐mediated apoptosis by downregulation of *c‐MYC* transcription and concurrent genomewide downregulation of *c‐myc* target genes in MM cells.^111^, ^112^ The BRD inhibitor CPI203 overcame melphalan^113^ and bortezomib resistance of MM cells in vitro,^114^ and in combination treatments, CPI203 synergized with bortezomib and melphalan as well as lenalidomide and dexamethasone regimens to induce apoptosis of MM cell lines.^114^, ^115^ Although none of these studies measured the effects of BRD inhibitors on development of MM bone lesions, Diaz and colleagues^115^ tested the effectiveness of the BRD inhibitor CPI203 on primary patient CD138+ MM cell survival in cocultures with BMSCs. In these experiments, CPI203 prevented BMSC‐mediated protection from the cytotoxic effects of the drug as well as the increased proliferation of MM cells usually found in BMSC cocultures. Interestingly, BETi suppressed chondrocyte differentiation in vitro and reduced bone growth in vivo in a zebra fish model.^116^ BRD4 binds to and upregulates expression of OB‐specific enhancers and matrix‐specific genes during lineage commitment during OB differentiation. Disturbances of BRD4 function negatively affect OB differentiation both during early commitment and later stages of mineral deposition.^117^ JQ1, a bromodomain inhibitor, was an effective treatment for primary osteosarcoma tumors in vivo.^118^ By inhibiting c‐*MYC* and *RUNX2* expression, JQ1 reduced both OB differentiation and primary bone tumor development.^118^ Collectively, these studies suggest that BET inhibitors may be valuable for treating certain malignancies and osteoblastic‐cancers, but their use in osteolytic diseases, including MMBD, may be limited because of their deleterious effects on osteogenic differentiation, which could prevent healing of bone lesions.

Several classes of small molecules targeting the histone methyltransferase EZH2 have been developed, and three EZH2 inhibitors (tazemetostat [EPZ‐6438], GSK2816126, and CPI‐1205) have moved into phase 1/phase 2 clinical trials in patients with diffuse large B‐cell and follicular lymphomas and genetically defined solid tumors.^119^ EZH2 inhibitors exhibit strong anti‐MM effects alone or in combination with conventional treatments and other types of epigenetic inhibitors. Abnormal EZH2/H3K27me3 activity has been implicated in the pathogenesis of MM, and the degree of EZH2 overexpression correlates with the aggressiveness of MM subtypes and poor prognosis in MM patients.^120^, ^121^ Homozygous mutations of EZH2 were described in myelodysplastic/myeloproliferative neoplasms^122^ but not in MM cell lines or primary patient cells.^123^ However, a subgroup of patients (∼15%) have overexpression of oncogenic methyltransferase MMSET in MM cells as a result of the t(4;14) translocation that creates the juxtaposition of the *MMSET* gene to the immunoglobulin heavy‐chain enhancer locus.^123^, ^124^ MMSET catalyzes dimethylation of H3K36 (H3K36me2), and its upregulation causes a global increase and redistribution of H3K36me3 modification across the genome.^125^ The increased H3K36me3 resulting from deregulated MMSET expression decreases the amount of global H3K27me3 marks in MM cells. Popovic and colleagues^124^ reported that in MMSET‐overexpressing cells, interplay between elevated H3K36me3 marks and EZH2 binding across the genome changes the global chromatin distribution of repressive H3K27me3 marks, which becomes enriched on selective promoters of MM genes associated with lymphoid biology, germinal center B cells, and downstream targets of c‐MYC. This aberrant hypermethylation of H3K27 at specific oncogenic loci in MM cells is associated with an increased sensitivity to EZH2 inhibitors.^124^ Similarly, deregulation of genomic H3K27me3 levels due to mutations/deletions of histone H3K27 demethylase *UTX* have been reported in several malignancies and occur in up to 10% of MM cases.^126^ Loss of *UTX* caused increased MM cell sensitivity to EZH2 inhibition in vitro and in mouse models of MM in vivo. Loss of *UTX* results in changes in the distribution of H3K27me3 and H3K27ac, which lead to deactivation of IRF4 and *c‐MYC* gene expression, ultimately promoting proliferation, clonogenicity, adhesion, and tumorigenicity of MM cells.^127^ EZH2 can regulate genes and miRNAs involved in stemness, growth, survival, differentiation, and angiogenesis,^(128,129)^ as well as adherence and epithelial‐mesenchymal transition in MM cells.^130^ Inhibition of EZH2 induced apoptosis in a variety of MM cell lines,^29^, ^128^, ^129^, ^131^ inhibited proliferation of MM cells with stem cell–like characteristics,^132^ and reduced subcutaneous growth of MM cell xenografts in mice.^130^ Dimopoulos and colleagues^133^ reported that the combination of 5‐azacitidine and EPZ‐6438 resensitized IMiD‐resistant OPM2 and NCI‐H929 human MM cells to lenalidomide and pomalidomide treatment. Dual inhibition of EZH2 and EZH1 with UNC1999 sensitized MM cells to proteasome inhibitors,^134^ and long‐term administration of a novel dual EZH2/1 inhibitor OR‐S1 eradicated minimal residual disease from the bone marrow in an orthotopic MM model and reduced immunoglobulin serum levels in a MM patient‐derived xenograft mouse model.^135^ Inhibition of EZH2 has been associated with osteo‐protective effects and positively regulating osteogenic differentiation. The EZH2i, GSK126, increased bone density in wild‐type adult mice and estrogen‐deficient mice after bilateral ovariectomy (OVX), an in vivo model of postmenopausal osteoporosis.^66^, ^69^, ^136^ Bone‐protecting effects of EZH2i were also observed by Jing and colleagues,^81^ who found that in vivo administration of DZNep enhanced osteogenic differentiation of BMSCs. Currently, there are no data evaluating the effects of EZH2 inhibition on bone restoration in MM disease models. A recent study by Adamik and colleagues^15^ showed that GSK126 rescued osteogenic differentiation of both MM‐exposed pre‐OBs and primary patient‐derived MM‐BMSCs in vitro. We recently found that GSK126 synergizes with bortezomib to induce anti‐MM effects in a 3D model of MM.^137^

PRC1 complexes monoubiquitinate histones (H2AK119ub) and cooperates with PRC2 to silence gene transcription.^138^ The primary core components of the PRC1 complex are CBX, RING1, PHC, BMI1, and RYBP/YAF2. Polycomb ring finger BMI1 is an indispensable subunit of PRC1, and its overexpression correlates with disease progression and therapy failure in many human cancers including MM.^139^, ^140^ BMI1 inhibition, using a small molecule inhibitor, PTC209, induced apoptosis in MM cell lines and primary MM cells in vitro.^140^ PTC209 exhibited synergistic and additive anti‐MM effects when combined with pomalidomide and carfilzomib,^141^, ^142^ as well as EZH2 and BET‐targeting epigenetic inhibitors.^129^ Further, BMI1 shRNA was effective in reducing tumor growth of MM xenografts in mice.^140^ Anti‐MM activity of PTC209 was significant even in the presence of MM growth factors insulin‐like growth factor 1 (IGF1) and IL6 as well as in cocultures with BMSCs.^142^ In the same study, PTC209 impaired OB formation in a dose‐dependent manner. Additional experiments showed that elevated expression of Dickkopf‐1 was responsible for the decrease in OB differentiation.^142^ Several studies demonstrated that BMI1 is indispensable for osteogenic differentiation.^143^, ^144^ Seo and colleagues^143^, ^144^ found that BMI1 cooperated with SOX2 to maintain self‐renewal and pluripotency of OB progenitors. BMI1 expression also increases during osteogenic differentiation of human adipose derived stem cells (hASCs) in vitro. Overexpression of BMI1 enhanced osteogenic differentiation of hASCs and increased BMP2 and WNT11 expression.^145^ BMI1 knockout mice have skeletal growth retardation, with decreased trabecular bone volume and bone mineral density.^146^ Further, BMI1‐deficient mice displayed exhaustion of the mesenchymal stem cell pool as well as impaired OB differentiation markers and mineral deposition rate that correlated with increased PPARγ expression and the number of bone marrow adipocytes.^146^ Consistent with BMI1's potential involvement in the osteo‐adipogenic switch, knockdown of BMI1 resulted in upregulation of PPARγ and blocked osteogenesis of human embryonic and induced pluripotent mesenchymal stem cells.^143^ In summary, although unexplored in the context of MMBD, increasing evidence shows that BMI1 is a critical modulator of proliferation and self‐renewal of mesenchymal stem cells. Because inhibition of BMI1 has adverse effects on skeletal progenitors, its use in MMBD may be limited.

LSD1 regulates a broad spectrum of biological processes, including maintenance of stemness and oncogenic gene programs during cancer progression.^147^ LSD1 is significantly overexpressed in patients with symptomatic MM and plasma cell leukemia.^148^ LSD1 can demethylate H3K4me2 and/or H3K9me and act as a transcriptional co‐repressor or co‐activator depending on the substrate recognition site.^147^ Several reports indicate that inhibition of LSD1 has anti‐MM effects.^148^, ^149^ LSD1 knockdown enhanced the cytotoxicity of HDACi (SAHA, LBH589) and significantly reduced expression of surface adhesion proteins, which diminished migration and invasion of MM1.S MM cells.^148^ Escoubet‐Lozach and colleagues^149^ showed that pomalidomide and lenalidomide facilitated re‐repression of the p21WAF1 promoter through an LSD1‐dependent mechanism and induced cell cycle arrest in Burkitt's lymphoma and MM cell lines in vitro. Upregulation of LSD1 protein along with downregulation of JMJD2B expression by a plant extract, triptolide, has also been shown to cause cell‐cycle arrest and apoptosis of RPMI8226 MM cells.^150^ Several studies reported that LSD1 inhibits osteogenic and supports adipogenic differentiation.^91^, ^92^, ^93^ LSD1 inhibitors, pargyline and CBB1007, have been shown to enhance alkaline phosphatase activity and extracellular matrix mineralization by hASCs without apparent cellular toxicity.^151^, ^152^ Further analysis showed that LSD1i rescued osteogenic differentiation by enhancing the dimethylation level of H3K4 at the promoter regions of osteogenesis‐related genes.^151^, ^152^ Experiments using in vivo collagen scaffolds infused with hASCs implanted subcutaneously in nude mice showed that scaffolds with LSD1‐knockdown hASCs exhibited increased density and ossification compared with scrambled controls.^152^ Further, treatment with the LSD1 inhibitor pargyline helped restore the osteogenic capacity of ex vivo expanded BMSCs from aged osteoporotic mice and/or OVX mouse models.^151^ Our results demonstrated that MM cocultured with BMSCs enhanced the recruitment of LSD1 to epigenetically suppress *Runx2* expression and the differentiation potential of OB progenitors.^15^ Results to date suggest that LSD1i may be a valuable treatment strategy in MM; however, further in vitro and preclinical in vivo studies of MM are needed to demonstrate its efficacy for MMBD.

## Conclusion

Oncogenic events such as genomic mutations, deletions, and recurrent chromosomal translocations often occur in MM.^153^ DNA methylation, histone modifications, or abnormal expression of several classes of non‐coding RNAs are emerging as underlying epigenetic mechanisms that contribute to the oncogenic transformation that underlies the pathogenesis and progression of MM.^24^, ^154^ Although the primary focus of myeloma research has been genomic and epigenetic alterations in MM cells, tumor‐associated epigenetic transformations in the supportive cellular bone compartments are largely unexplored. Results from recent studies reviewed in this work demonstrate that deregulated epigenetic modifiers play a critical role in establishment and maintenance of the persistent pathological alterations in MM‐BMSCs that occur in MM. We speculate that MM cell exposure hijacks the epigenetic plasticity of pluripotent BMSCs and reprograms their fate toward adipogenesis, thereby suppressing their osteogenic capacity.

The realization that epigenetic mechanisms drive oncogenic transformation, clonal heterogeneity, and the response and adaptation of cancer cells to treatment opened a new frontier for development and the use of small molecule epigenetic inhibitors as novel treatments for malignancies. However, in terms of myeloma research, the effects of these molecules are often studied on MM cells cultured in the absence of surrounding bone environment, both in vitro as well as in vivo subcutaneous tumor models. Given the vital importance of microenvironmental support for tumor growth and chemo‐resistance, it is imperative that more MM studies are executed in the context of the myeloma bone setting. This will also yield valuable information pertaining to bone cell responses to the treatments. Because epigenetic mechanisms are reversible forms of gene regulation, the use of these agents can be modulated and fine‐tuned to achieve the best bone anabolic effects and minimize the risk of side effects. In addition to their use as single agents, the multifactorial use of epigenetic inhibitors in combination with conventional drugs opens up yet another frontier of therapeutic intervention against MMBD.

## Disclosures

All of the authors state that they have no conflict of interest.
